# Supplementary material for: A Global Screen for Assembly State Changes of the Mitotic Proteome by SEC-SWATH-MS
Source: Cell Syst. 2020 Feb 26;10(2):133–155.e6. doi: 10.1016/j.cels.2020.01.001 (PMC7042714; doi:10.1016/j.cels.2020.01.001)

Q8NFP9 | NBEA_HUMAN | NBEA BCL8B KIAA1544 LYST2
Monomer MW [kDa]: 327.822 Monomer expected elution fraction: 30

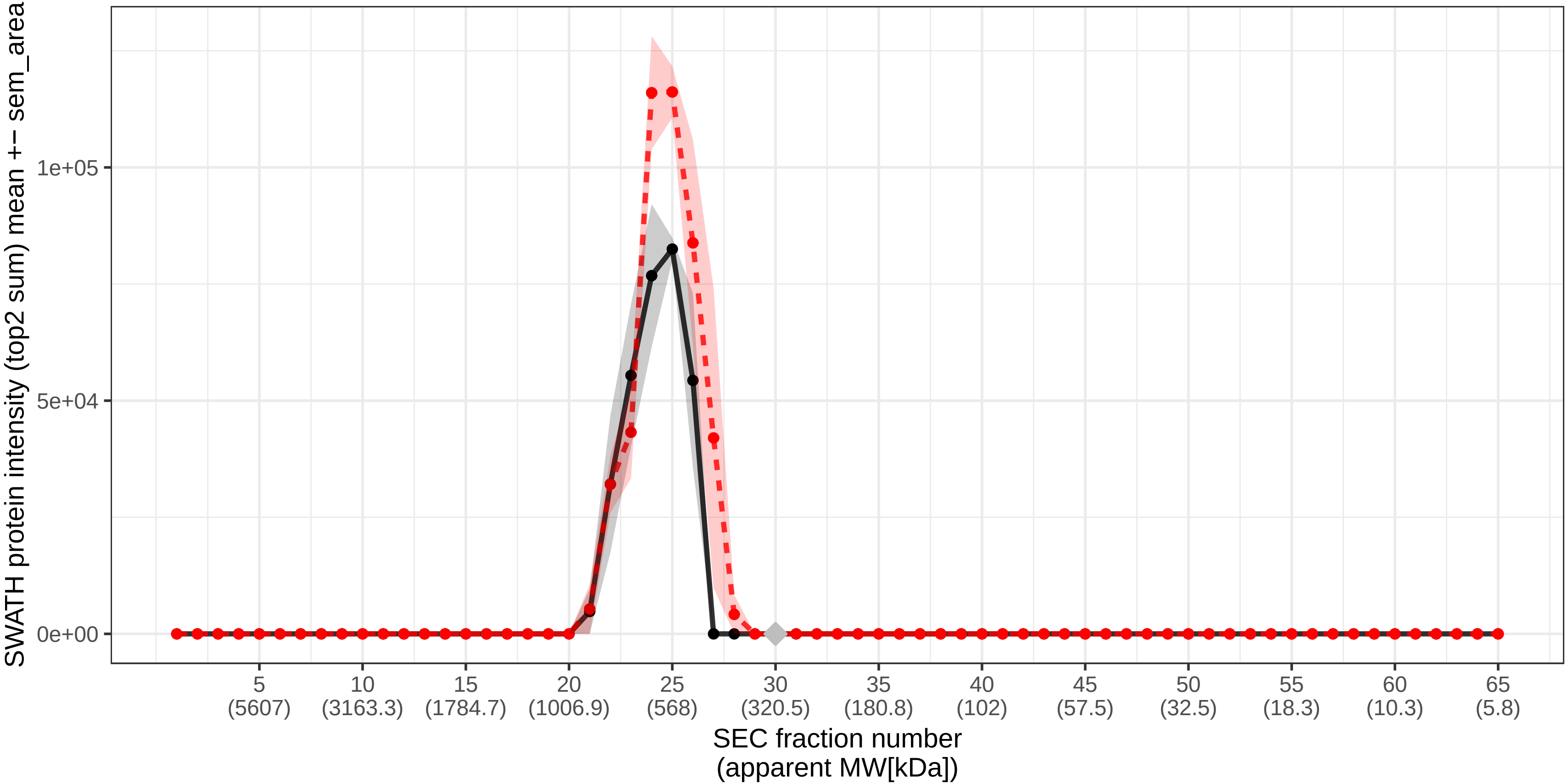

Supplement: Data S1. SEC-SWATH-MS Protein Chromatograms, Related to Figure 1 [file mmc6.zip › SECchrom_Q8NFP9_NBEA_HUMAN_NBEA_BCL8B_KIAA1544_LYST2.pdf]
